# Study on Network Pharmacological Analysis and Preliminary Validation to Understand the Mechanisms of Plantaginis Semen in Treatment of Gouty Nephropathy

**DOI:** 10.1155/2020/8861110

**Published:** 2020-11-27

**Authors:** Hong Zhao, Qi Gao, Ling-zhou Kong, Wei-wei Tang, Ying-ying Jiao, Yu-liang Wang, Zhang Yu, Yao Feng

**Affiliations:** ^1^College of Pharmacy, Jiamusi University, Jiamusi 154007, China; ^2^The First Affiliated Hospital of Jiamusi University, Jiamusi 154007, China

## Abstract

Plantaginis Semen (PS) has been used to promote diuresis and clear away dampness. Recent reports have shown that PS can be used to treat gouty nephropathy (GN). However, the action and mechanism of PS have not been well defined in treating GN. The present study aimed to define the molecular mechanisms of PS as a potential therapeutic approach to treat GN. A combination of network pharmacology and validation experiments in GN is used to understand the potential mechanism. Information on pharmaceutically active compounds in PS and gene information related to GN was obtained from public databases. The compound target network and protein-protein interaction network were constructed to study the mechanism of action of PS in the treatment of GN. The mechanism of action of PS in the treatment of GN was analyzed via Gene Ontology (GO) biological process annotation and Kyoto Gene and Genomics Encyclopedia (KEGG) pathway enrichment. Validation experiments were performed to verify the core targets. The GN rat model was prepared by the method of combining yeast and adenine. Hematoxylin-eosin (HE) staining was used to observe the morphology of renal tissue in rats. ELISA was applied to detect TGF-*β*1, TNF-*α*, and IL-1*β* levels in renal tissue. The expressions of TGF-*β*1, TNF-*α*, and IL-1*β* were determined using immunohistochemistry. Through the results of network pharmacology, we obtained 9 active components, 118 predicted targets, and 149 GN targets from the public database. Based on the protein-protein interaction (PPI), 26 hub genes for interaction with PS treating for GN were screened, including MMP9, TNF, IL1*β*, and IL6. The enrichment analysis results showed that the treatment of GN with PS was mainly involved in the TGF-*β*1 signaling pathway, MAPK signaling pathway, TNF signaling pathway, NF-*κ*B signaling pathway, and PI3K Akt signaling pathway. Validation experiment results showed that PS could reduce the content of urinary protein and UA and deregulate the expression of TGF-*β*1, TNF-*α*, and IL-1*β* in the treatment of GN. The molecular mechanism of PS in the treatment of GN indicated the synergistic features of multicomponent, multitarget, and multipathway of traditional Chinese medicine, which provided an essential scientific basis for further elucidating the mechanism of PS in the treatment of GN.

## 1. Introduction

Gouty nephropathy (GN) is mainly caused by the deposition of uric acid salt in the blood concentration of the supersaturated state. It is a kind of disease with uric acid stone and interstitial nephritis as the main pathological changes. As the disease progresses, it can eventually lead to kidney failure [1]. It may threaten the life and health of patients in some cases. The GN patients mainly include hyperpimelic middle-aged men and menopausal women, and it is tendency to become younger [2]. The development cycle of GN is relatively slow. In early diagnosis and appropriate treatment, it is essential to improve renal function and stabilize the disease. At present, corticosteroids and nonsteroidal anti-inflammatory drugs (NSAIDs) have been applied to treat GN. However, presently available therapies are not more effective and often have adverse effects [3, 4]. Literature reported that an increasing number of TCMs have the protective effect of the kidney, and then, increasing attention has been paid to TCM in treatment of nephropathy because it has a good and mild activity.

Plantaginis Semen (PS) is a traditional Chinese herbal medicine with diuretic, anti-inflammatory, and immune regulation effects [5–7]. After extensive research, it was found that PS contains a variety of active components, such as polysaccharides, iridoids, triterpenes, flavonoids, and alkaloids, and many of these ingredients are verified to have certain effects on xanthine oxidase (XOD) activity and uric acid excretion [8, 9]. Under the guidance of traditional Chinese medicine theory, PS is a diuretic drug, and it has the characteristics of mild drug and little side effects. Many prescriptions composed of PS were used for treating GN. Therefore, PS has an important value in the development of GN.

Network pharmacology is based on the theory of system biology and multidirectional pharmacology. It usually uses a complex network to explore the mechanism of action of drugs. It is suitable for studying the relationship between multicomponents and multitargets of traditional Chinese medicine (TCM) [10, 11]. The current study explored the mechanisms of PS for treating GN by applying network pharmacology, to provide credible evidence for the mechanism of PS treating GN.

## 2. Materials and Methods

### 2.1. Reagents and Chemicals

Plantaginis Semen was purchased from Tongrentang Chinese Medicine (Beijing, China). Adenine, yeast powder, and allopurinol tablets were purchased from Sigma-Aldrich Co., Ltd. (USA), Chengxin Biotechnology Co., Ltd., and Linfen Qilin Pharmaceutical Co., Ltd., respectively. The detection kits of urine protein, UA, BUN, Cr, and enzyme-linked immune sorbent assay (ELISA) for the measurement of TGF-*β*1, TNF-*α*, and IL-1*β* and CBB were purchased from the Nanjing Jiancheng Bioengineering Institute (Nanjing, China). Antibodies TGF-*β*1, TNF-*α*, and IL-1*β* were provided by Beijing Bioss Biotechnology Co., Ltd. (Beijing, China).

### 2.2. Collection and Screening of Active Components

The TCMSP (http://tcmspw.com/tcmsp.php) is used to search for the useful active components of PS [12]; the key word is “Plantaginis Semen,” and the screening condition is “OB ≥ 30%, DL ≥ 0.18.” OB (oral bioavailability) refers to the speed and extent of the drug entering the body circulation after oral absorption [13]. DL (drug-like index) is used to reflect the similarity of specific groups in the compound with known drugs degree [14]. Oral bioavailability and drug-like properties play essential roles in the study of the TCM active ingredient.

### 2.3. Intersection Analysis of PS and GN-Related Targets

The predicted targets related to PS active ingredient were input into the UniProt database (https://www.uniprot.org/) for screening [15].

The OMIM (http://www.omim.org/) [16] and GeneCards (https://www.genecards.org/) [17] databases were used to collect GN-related disease targets with “Gouty nephropathy” as the keyword. The unnecessary duplicate targets were deleted.

In order to understand the connection between drugs and disease, the PS target and disease target were entered into Venny website (https://bioinfogp.cnb.csic.es/tools/venny/index.html), and the intersection target was obtained.

### 2.4. Protein-Protein Interaction Data

The intersection target was uploaded to STRING 11.0 (https://string-db.org/) [18], and the irrelevant nodes in the PPI network were hidden. The results were exported in PNG and TSV formats. The TSV results were put into the Cytoscape 3.7.1, and the “network analyzer” plug-in was used to visualize.

### 2.5. Pathway and Functional Enrichment Analysis

The intersection target was imported into the DAVID database (https://david.ncifcrf.gov/) [19] for Gene Ontology (GO) and Kyoto Encyclopedia of Genes and Genomes (KEGG) pathway enrichment analysis.

### 2.6. Animals

A total of 60 male SD rats, at 10 weeks of age, were purchased from the Animal Experiment Center of Jiamusi University. The animals were housed in polyacrylic cages (five mice per cage) under well-controlled conditions (room temperature of 22 ± 1°C, relative humidity of 50% ± 5%, and 12 h : 12 h light/dark cycle) with food and water ad libitum. The protocol was approved by the Committee on the Ethics of Animal Experiments of Jiamusi University, China, and the approval number was JMSU-231.

### 2.7. Establishment of the GN Rat Model

The GN rat model was prepared by the method of combining yeast and adenine. The suspension of intragastrical gavage (IG) was prepared by 0.5% sodium carboxymethyl cellulose (CMC-Na) solution and adenine. At the same time, the yeast dry powder was mixed into the regular feed of rats, and the daily intake of yeast was controlled to 10 g·kg^−1^.

Rats were randomly divided into 6 groups (*n* = 10): the control group, model group, positive control group (allopurinol 50 mg·kg^−1^·d^−1^),PS high-dose group (1.62 g·kg^−1^·d^−1^, PS-HD),PS medium-dose group (0.81 g·kg^−1^·d^−1^,PS-MD), and PS low-dose group (0.27 g·kg^−1^·d^−1^,PS-LD). Control group rats were given 0.5% CMC-Na solution in a volume of 100 mg·kg^−1^. The others were administered with combined adenine (100 mg·kg^−1^) and 0.5% CMC-Na (100 mg·kg^−1^) by IG once daily at 8 : 00 a.m. for 28 days. At the beginning of modelling, the rats of each group were separately given PS decoction or allopurinol every afternoon. At 18 consecutive days, the serum samples from the rats' orbital veins were taken for the measurement of contents of UA, Cr, and BUN. The animal model was established successfully if the contents of UA, Cr, and BUN increased significantly. With the prolongation of the modelling time, the rats in the model group showed signs of hair loss, lethargy, arched back, and weight loss. Each administration group was lighter than the model group, but not as healthy as the normal control group.

### 2.8. Pharmacological Experiment

#### 2.8.1. Detection of 24 h Urine Protein

The 24 h urine was collected by the metabolic cage method. The 24 h urine protein content was determined by using the urine protein quantitative test box (CBB) in the experiment and on the 7th, 14th, 21st, and 28th days.

#### 2.8.2. Biochemical Parameters

On the 28th day, rats were anaesthetized with 20% urethane. The blood samples were taken from the abdominal aorta, and serum was separated by centrifugation at 3000 r·min^−1^, 4°C for 10 min. The contents of UA, BUN, and Cr in serum were determined by using an automatic biochemical analyzer.

#### 2.8.3. Renal Weight Index

On the 28th day, the rats were killed immediately, and the kidney index was calculated.

(1) $$\begin{array}{c} \text{Kidney index}=\text{double kidney weight}/\text{rat weight}\times 100\text{\%.} \end{array}$$

#### 2.8.4. Detection of Related Enzymes in Serum

According to the instructions of the xanthine oxidase (XOD) kit, the content of XOD in serum was detected by colorimetry.

#### 2.8.5. Histomorphology Observation

The renal tissue was fixed with 10% formaldehyde and, then, embedded in paraffin. Each specimen was cut into 4 *μ*m sections and mounted on APES-coated glass slides. Sections were deparaffinized in xylene, rehydrated in decreasing concentrations of alcohol in water, and then, used for HE staining. The pathological changes of renal tissue were observed under an optical microscope (×400).

#### 2.8.6. Detection of Specific Antibody

Serum TGF-*β*1, TNF-*α*, and IL-1*β* levels were determined by using the ELISA kits.

#### 2.8.7. Semiquantitative Analysis of Immunohistochemical Staining

The expressions of TGF-*β*1, TNF-*α*, IL-1*β*, and IL-6 in renal tissue were detected by immunohistochemistry.

After dewaxing and incubation, the slices were added with citrate buffer solution (PH = 6), heated in a microwave oven, and cooled to room temperature naturally. The first antibody and the second antibody were added successively and incubated at 37°C for color development. They were dehydrated and transparent, sealed, and observed under the optical microscope.

The images were captured with the Motic 3000 (400×). The optical density values of positive reactions in the visual fields were counted by using the Image-Pro Plus 6.0 pathological image analysis system. The integrated optical density (IOD) of positive reactions represented the relative protein expression.

### 2.9. Statistical Analysis

All values were expressed as mean ± SD. A one-way analysis of variance (ANOVA) was used to detect the statistical significance followed by post hoc Dunn's multiple comparisons test by EXCEL, and *P* < 0.05 was considered as statistically significant.

## 3. Results

### 3.1. Screening of PS and GN-Related Targets

Nine active components were obtained from PS, and 118 potential targets of PS were obtained from these nine components (Table 1).

All the targets related to GN were searched in the OMIM and GeneCards database, and 149 GN-related targets were obtained.

**Figure 1:**
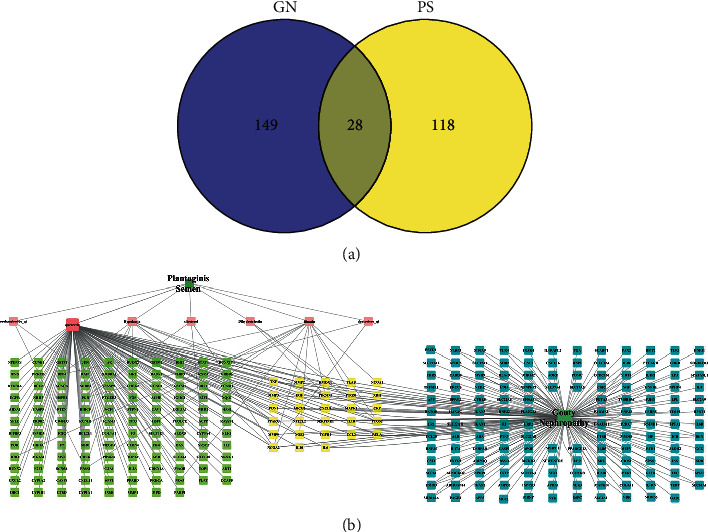
(a) Venn diagram of PS and GN-related targets. (b) PS active compound target-GN-related targets network.

In order to clarify the relationship between PS and the GN-related target, 28 intersection targets of PS and GN were obtained (Figure 1).

### 3.2. Protein-Protein Interaction Analysis

**Figure 2:**
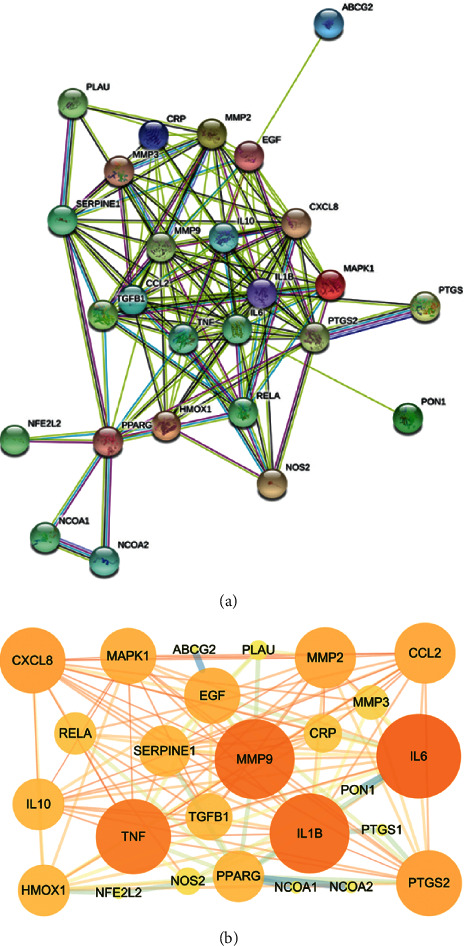
PPI network of intersection targets.

Based on the results of PNG and TSV, the PPI network (Figure 2(a)) contains 28 nodes and 138 edges, and the average number of nodes is 9.36. In order to study the relationship between target proteins, TSV was put into the Cytoscape 3.7.1 for visualization and plotted according to the degree value. The TGF-*β*1, TNF-*α*, IL-1*β*, IL-6, MMP9, and TGF-*β*1 which had higher degree value may be the key targets for the treatment of GN by active ingredients of PS, as shown in Figure 2(b).

### 3.3. Enrichment Analysis of Related Pathways and the Biological Process

**Figure 3:**
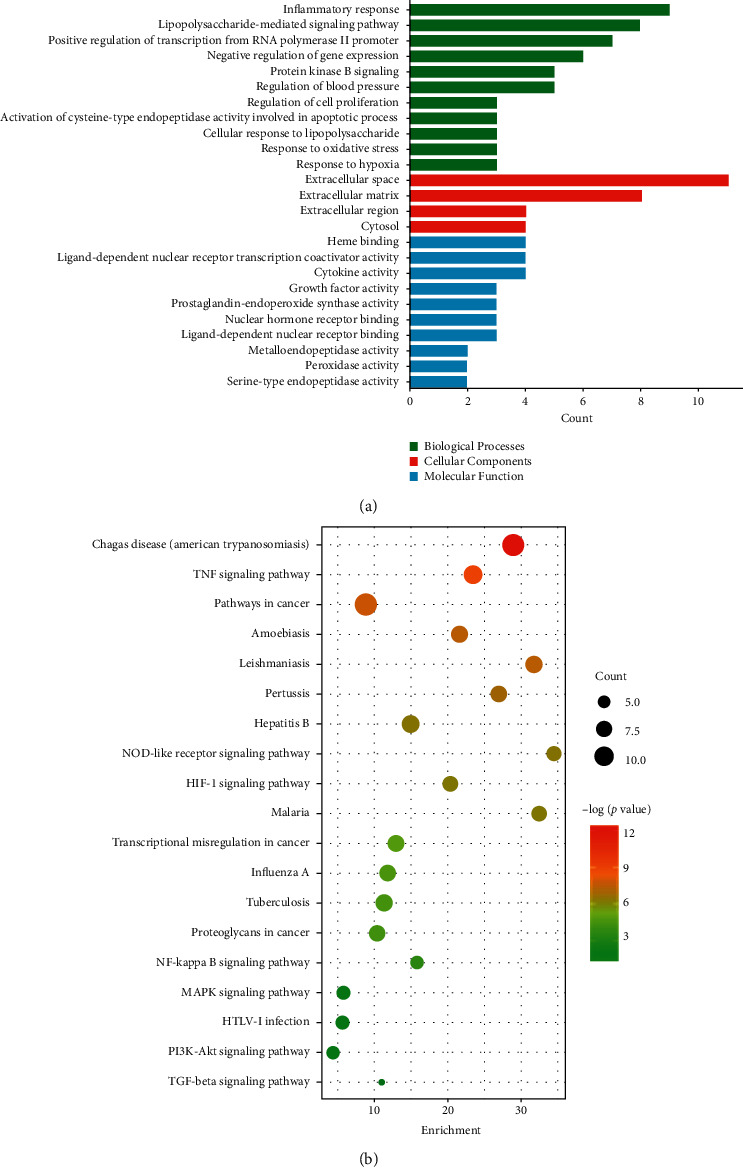
(a) GO enrichment analysis of key targets; (b) KEGG pathways enrichment analysis of key targets.

The DAVID database was used to analyze the GO function analysis and KEGG pathway enrichment analysis of the target genes of PS treating GN. The functional distribution of 58 targets was explored by GO functional analysis, of which 40 entries are related to biological processes (BP), including inflammatory response, response to oxidative stress, and response to hypoxia. Fourteen items are related to molecular function (MF), including cytokine activity and heme binding, and 21 cell components (CC) entries include the extractive matrix and extracellular space (Figure 3(a)). The result indicated that PS might affect the occurrence and development of GN by regulating the abovementioned biological processes.

To determine the relevant signaling pathways involved in the GN effect of PS, we conducted pathway enrichment analysis using KEGG pathways. A total of 28 targets obtained 90 KEGG signaling pathways (*P* < 0.01), including the MAPK signaling pathway, TNF signaling pathway, TGF-*β* signaling pathway, NF-*κ*B signaling pathway, and PI3K Akt signaling pathway, as shown in Figure 3(b). The results showed that the active target of PS could play a synergistic therapeutic role by regulating multiple pathways.

### 3.4. The Effect on 24 h Urine Protein

**Figure 4:**
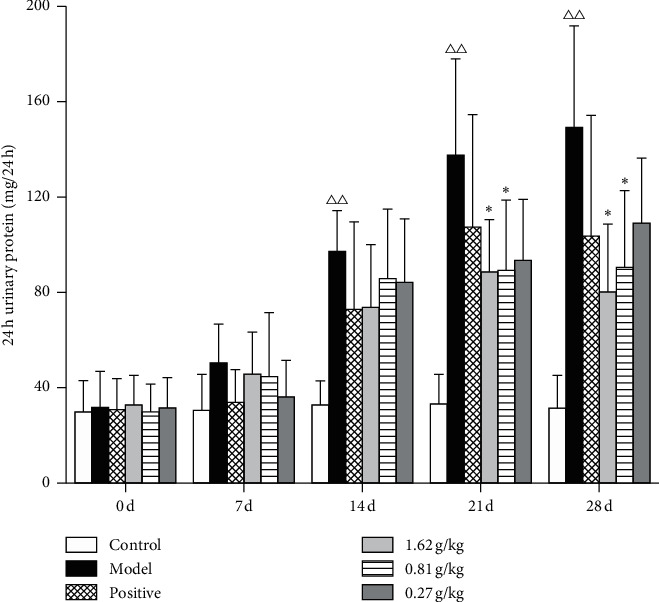
The effect on 24 h urinary protein in GN rats. ^Δ^*P* < 0.05, ^ΔΔ^*P* < 0.01, compared with the control group; ^*∗*^*P* < 0.05, ^*∗∗*^*P* < 0.01, compared with the model group.

There was no significant difference in 24 h urine protein among all groups (*P* > 0.05) on the 7th day. The 24 h urinary protein in the model group rats had increased (*P* < 0.01) compared with that in the control group rats on the 14th and 28th day; the 24 h urinary protein in the PS-HD and PS-MD group rats was decreased (*P* < 0.05), compared with that in the model group rats, as shown in Figure 4. These results indicate that PS can reduce the 24 h urinary protein content of GN rats.

### 3.5. The Effect on Serum Biochemical Indexes

**Figure 5:**
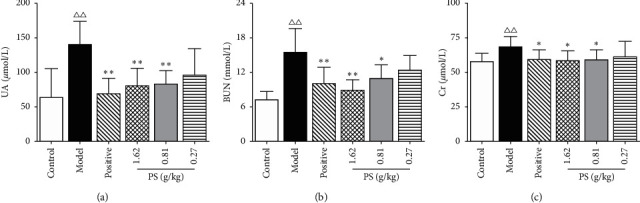
Effect on serum biochemical indexes in GN rats.

Compared with the control group, the levels of the UA, BUN, and Cr were increased (*P* < 0.01) in the model group. Compared with the model group, the levels of UA and BUN in the allopurinol group and PS-HD group rats were decreased (*P* < 0.01). The level of UA in the PS-MD group was decreased (*P* < 0.01), and the level of BUN was decreased (*P* < 0.05). Simultaneously, the level of Cr in the allopurinol group, PS-HD, and PS-MD group was decreased (*P* < 0.05), as shown in Figure 5. The results showed that PS could reduce the levels of UA, BUN, and Cr in the serum of GN rats.

### 3.6. The Effect on the Kidney Weight Index

**Figure 6:**
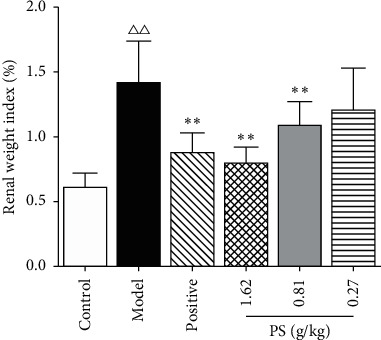
Effect on the kidney weight index in GN rats.

The kidney weight index in the model group had increased (*P* < 0.01) compared with the control group. Compared with the model group, the kidney weight index of the PS-MD group was decreased (*P* < 0.05); the kidney weight index of the positive group and PS-HD group was decreased (*P* < 0.01), as shown in Figure 6. The results showed that PS could reduce the kidney weight index of GN rats.

### 3.7. The Effect on XOD in Serum of GN Rats

**Figure 7:**
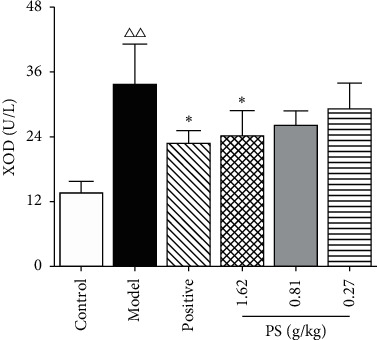
Effect on XOD in GN rats.

Compared with the control group, the content of XOD in serum of the model group was increased (*P* < 0.01). Compared with the model group, the content of XOD in the PS-HD group was decreased (*P* < 0.05), as shown in Figure 7. The results showed that PS could reduce XOD in serum of GN rats.

### 3.8. The Effect on Renal Morphology of GN Rats

In the control group, the renal tissue structure and glomerular morphology were normal; the renal tubular epithelial cells were arranged orderly and uniform in size, without inflammatory cell infiltration and pathological changes.

**Figure 8:**
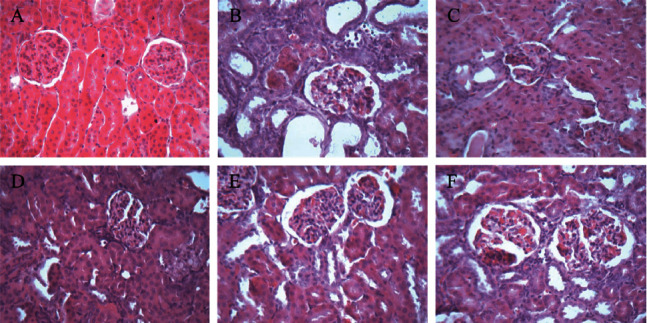
Renal histopathology HE staining (×400). (a) Control. (b) Model. (c) Positive. (d) PS-HD group. (e) PS-MD group. (f) PS-LD group.

Compared with the control group, there were more significant pathological changes in the renal tissue of the model group, the number of glomeruli decreased, the vascular atrophy or even disappeared, the edema of renal tubular epithelial cells were prominent, and noticeable yellow-brown urate crystals were visible. There were a large number of inflammatory cells infiltrated in the renal interstitium, which can lead to renal interstitial fibrosis (Figure 8).

Compared with the model group, the pathological changes of renal tissue in each treatment group were alleviated to some extent, the yellow-brown urate crystals of renal tubules were reduced, inflammatory cell infiltration was less, and the degree of renal interstitial fibrosis was slight. The ameliorating effect of the PS-HD group and allopurinol group was significant (Figure 8).

### 3.9. The Effects on Potential Targets

**Figure 9:**
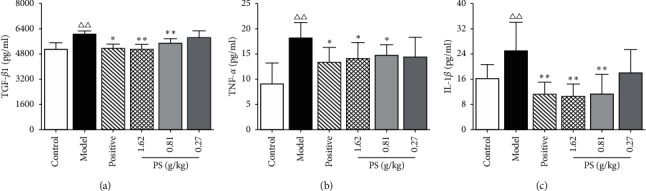
Effect on the expression of inflammatory cytokines in GN rats.

Compared with the control group, the levels of TGF-*β*1, TNF-*α*, and IL-1*β* in the model group were increased (*P* < 0.01). Compared with the model group, the levels of TGF-*β*1and IL-1*β* in the serum of PS-HD and PS-MD group rats were decreased (*P* < 0.01), and the level of TNF-*α* was decreased (*P* < 0.05), as shown in Figure 9. The results showed that PS could reduce the expression of TGF-*β*1, TNF-*α*, and IL-1*β* in the serum of GN rats.

### 3.10. Immunohistochemistry and Semiquantitative Analysis of Renal Tissue

**Figure 10:**
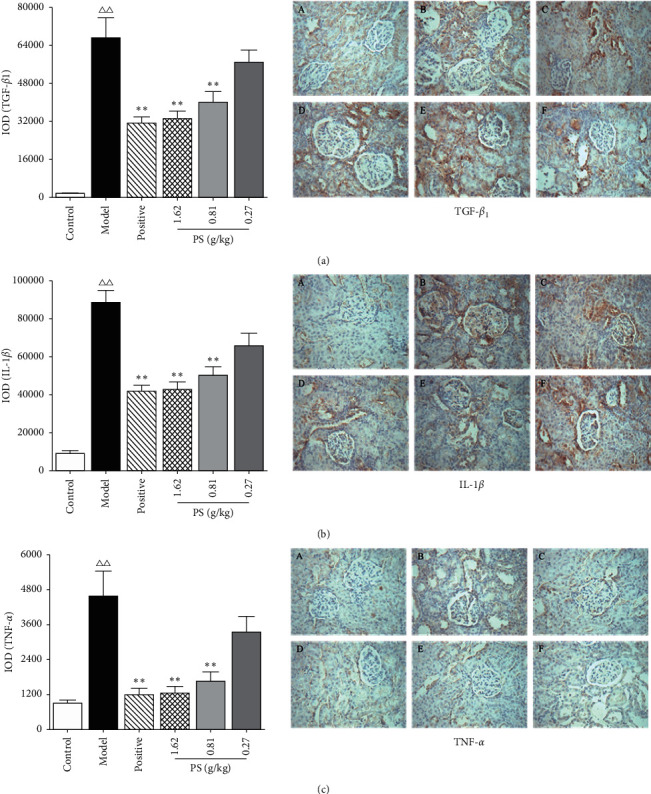
Effect on the expression of TGF-*β*1, TNF-*α*, and IL-1*β* in GN rats. (A) Control. (B) Model. (C) Positive. (D) PS-HD group. (E) PS-MD group. (F) PS-LD group.

Compared with the control group, the IOD of TGF-*β*1, TNF-*α*, and IL-1*β* in the model group rats was increased (*P* < 0.01). Compared with the model group, the IOD of TGF-*β*1, TNF-*α*, and IL-1*β* in the positive group, PS-HD, and PS-MD group was decreased (*P* < 0.01), as shown in Figure 10. The results showed that PS could significantly inhibit the expression of TGF-*β*1, TNF-*α*, and IL-1*β* protein in renal tissue of GN rats.

## 4. Discussion

The pathogenesis of GN is usually due to the disorder of purine metabolism, which generates very high production of uric acid or excessively little excretion of uric acid through the kidney, and further leads to the deposition of MSU crystals in the renal tubules and interstitium, ultimately resulting in tubular epithelial cell necrosis, tubular atrophy, and renal interstitial fibrosis [20, 21]. MSU crystal is considered to be one of the critical inducing factors of gout inflammation. It can activate macrophages, cause the production of inflammatory factors such as TGF-*β*1, TNF-*α*, IL-6, IL-1*β*, and oxidative stress, and finally, lead to tissue fibrosis [22–24]. Therefore, MSU crystal activation of inflammatory factors may lead to the development of GN.

According to the results of network pharmacology active components, quercetin, sitosterol, dinatin, dihydrotricetin, and hypolaetin are contained in PS. Quercetin has anti-inflammatory, oxidative stress, and immune regulation effects. It can reverse the deposition of the extracellular matrix of the renal tubular epithelium by inhibiting the expression of the p38MAPK signaling pathway, thus preventing the occurrence and development of renal fibrosis. Sitosterol has an anti-inflammatory effect similar to the hormone, which can block the release of inflammatory factors TNF-*α* and IL-6 and participate in the regulation of the NF-*κ*B signaling pathway. Dinatin has anti-inflammatory, antioxidant, antitumor, and other pharmacological activities, which can inhibit the expression of NLRP3 inflammatory bodies [25–27].

**Figure 11:**
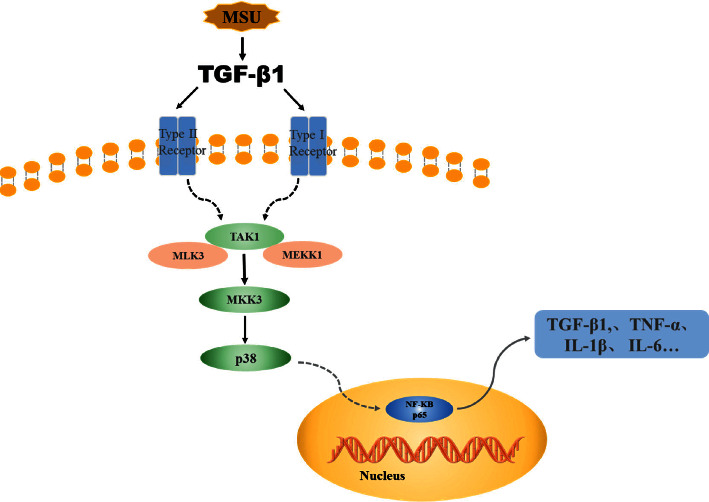
Overview of the potential mechanisms underlying the protective effects of PS on MSU-induced GN.

According to the experimental results of KEGG and PPI in network pharmacology and literature review, the TGF-*β*1/p38MAPK/NF-*κ*B signaling pathway plays a significant role in GN activated by MSU [28–30]. TGF-*β*1 is a multifunctional cytokine, which can activate the inflammatory signal pathway, promote the infiltration of inflammatory cells, and produce inflammatory factors, thus participating in the process of renal interstitial fibrosis. It is the key cytokine of renal interstitial fibrosis [31, 32]. The p38MAPK is an activated kinase, involving oxidative stress and inflammatory reaction, and can activate the downstream important signal factor NF-*κ*B [33, 34]. After the deposition of MSU crystals in the renal tubules or interstitium, TGF-*β*1 is stimulated to oversecrete, thus activating TAK1, promoting MKK kinase to activate p38MAPK phosphorylation. After being phosphorylated, p38MAPK can induce the high expression of the downstream NF-*κ*B signal pathway and, finally, promote the activation of inflammatory factors such as TNF-*α*, IL-1*β*, IL-6, and TGF-*β*1 and aggravate renal interstitial fibrosis and renal tissue inflammatory damage [35, 36], so inhibiting TGF-*β*1 may be the key of treating GN, as shown in Figure 11.

In order to verify the experimental results of network pharmacology, we used yeast and adenine to prepare the GN rat model and fed PS decoction for pharmacological verification. Gout nephropathy is usually characterized by increased serum UA and urinary protein secretion that can aggravate glomerulosclerosis and renal interstitial fibrosis. Therefore, the determination of UA and urinary protein is of great significance in the diagnosis of GN. The results of pharmacological experiments illustrate that PS could reduce the contents of urinary protein, UA, BUN, and Cr and activity of XOD. Uric acid was the product of purine metabolism, and XOD was the main enzyme of purine compounds metabolism in vivo. UA, BUN, and Cr are the main indicators of renal function, which reflect the uric acid content, glomerular filtration, and renal tubular reabsorption function. When renal parenchymal damage occurs, UA, BUN, and Cr levels increased. Therefore, PS may reduce the level of uric acid by mediating the activity of XOD, accelerate the increase of uric acid in the body, and finally, reduce the damage of renal tissue. The TGF-*β*1/p38MAPK/NF-*κ*B signaling pathway predicted was verified. Many inflammatory factors play a critical role in renal diseases, such as TGF-*β*1, TNF-*α*, and IL-1*β*; the activation and proliferation of intrinsic cells were resulted by them, and then, the occurrence and progression of renal diseases is aggravated. The results of pharmacological experiments showed that PS can decreased the contents of TGF-*β*1, TNF-*α*, and IL-1*β* in GN rats. It was preliminarily confirmed that PS could regulate the TGF-*β*1/p38MAPK/NF-*κ*B pathway and improve GN injury, which was consistent with the results predicted by network pharmacology.

## 5. Conclusions

In this study, the network pharmacology was used to analyze the mechanism and active ingredients of PS treating GN. The GN-related biological targets were measured by validation experiments. It was preliminarily confirmed that PS could inhibit the release and expression of inflammatory factors such as TGF-*β*1, TNF-*α*, and IL-1*β* by regulating the TGF-*β*1/p38MAPK/NF-*κ*B signaling pathway, to improve renal tissue injury, inflammatory factor infiltration, and renal fibrosis in GN rats. Due to the complexity of the renal fibrosis mechanism, this study aims to lay a foundation for further study of its mechanism.

## Figures and Tables

**Table 1:** Active components of Plantaginis Semen.

Mol. ID	Active components	OB (%)	DL
MOL001663	(4aS, 6aR, 6aS, 6bR, 8aR, 10R, 12aR, 14bS)-10-hydroxy-2, 2, 6a, 6b, 9, 9, 12a-heptamethyl-1, 3, 4, 5, 6, 6a, 7, 8, 8a, 10, 11, 12, 13, 14b-tetradecahydropicene-4a-carboxylic acid	32.03	0.76
MOL001735	Dinatin	30.97	0.27
MOL000359	Sitosterol	36.91	0.75
MOL005869	Daucostero_qt	36.91	0.75
MOL007813	Dihydrotricetin	58.12	0.28
MOL007819	Hypolaetin	33.24	0.28
MOL007835	Orobanchoside_qt	55.99	0.82
MOL007836	Plantaginin_qt	54.04	0.24
MOL000098	Quercetin	46.43	0.28

## Data Availability

The data that support the findings of this study are available from the first author upon reasonable request.
